# Self-reported practices among traditional birth attendants surveyed in western Kenya: a descriptive study

**DOI:** 10.1186/s12884-016-1007-8

**Published:** 2016-08-12

**Authors:** Sherri Bucher, Olive Konana, Edward Liechty, Ana Garces, Peter Gisore, Irene Marete, Constance Tenge, Evelyn Shipala, Linda Wright, Fabian Esamai

**Affiliations:** 1 Department of Pediatrics, Section of Neonatal-Perinatal Medicine, Indiana University School of Medicine, 699 Riley Hospital Drive, RR208, Indianapolis, IN 46202-5119 USA; 2 INSALUD, Guatemala City, Guatemala; 3 Department Child Health and Paediatrics, Moi University School of Medicine, Moi University, Eldoret, Kenya; 4 Moi Teaching and Referral Hospital, Eldoret, Kenya; 5 Center for Research for Mothers and Children, Eunice Kennedy Shriver National Institute of Child Health and Human Development, Bethesda, MD USA

**Keywords:** Traditional birth attendant, Maternal-newborn health, Delivery practices, Africa, Kenya, Health policy

## Abstract

**Background:**

The high rate of home deliveries conducted by unskilled birth attendants in resource-limited settings is an important global health issue because it is believed to be a significant contributing factor to maternal and newborn mortality. Given the large number of deliveries that are managed by unskilled or traditional birth attendants outside of health facilities, and the fact that there is on-going discussion regarding the role of traditional birth attendants in the maternal newborn health (MNH) service continuum, we sought to ascertain the practices of traditional birth attendants in our catchment area. The findings of this descriptive study might help inform conversations regarding the roles that traditional birth attendants can play in maternal-newborn health care.

**Methods:**

A structured questionnaire was used in a survey that included one hundred unskilled birth attendants in western Kenya. Descriptive statistics were employed.

**Results:**

Inappropriate or outdated practices were reported in relation to some obstetric complications and newborn care. Encouraging results were reported with regard to positive relationships that traditional birth attendants have with their local health facilities. Furthermore, high rates of referral to health facilities was reported for many common obstetric emergencies and similar rates for reporting of pregnancy outcomes to village elders and chiefs.

**Conclusions:**

Potentially harmful or outdated practices with regard to maternal and newborn care among traditional birth attendants in western Kenya were revealed by this study. There were high rates of traditional birth attendant referrals of pregnant mothers with obstetric complications to health facilities. Policy makers may consider re-educating and re-defining the roles and responsibilities of traditional birth attendants in maternal and neonatal health care based on the findings of this survey.

**Electronic supplementary material:**

The online version of this article (doi:10.1186/s12884-016-1007-8) contains supplementary material, which is available to authorized users.

## Background

In low- and middle-income countries, a large percentage of babies are born outside of health facilities. Many of these births are attended by unskilled community-based birth attendants [1]. The high rate of home deliveries conducted by unskilled birth attendants in resource-limited settings is believed to be a crucial underlying factor contributing to high rates of global maternal and newborn mortality [2, 3]. Approximately 90 % of maternal deaths and 98 % [4] of all perinatal mortality occurs in low and middle- income countries, where birthing outside of a health facility either alone, with untrained family members, or in the presence of unskilled birth attendants, is common.

There is continued debate within the international public health community regarding the most effective manner by which to increase the number of women who are attended by skilled birth attendants [5–8]. Ultimately, the most successful models by which to improve maternal and newborn health and reduce global rates of maternal and neonatal morbidity and mortality probably include a combination of strategies.

Traditional Birth Attendants (TBAs) have been a controversial and sometimes contentious issue in sub-Saharan Africa, with some countries instituting or debating a national ban on TBA activities [9, 10] In Kenya, the activities of TBAs have been discouraged by the Government for several years; women are encouraged to seek delivery in health facilities with the assistance of skilled birth attendants [11]. In 2005, the Kenyan government implemented a community midwifery program, but widespread scale-up has been limited by a number of factors, primarily poor funding [12]. While the overall proportion of births attended by skilled health professionals in Kenya has risen in recent years, from 44 to 62 %, with a concomitant increase in the proportion of deliveries that occur in a health facility (43 to 62 %) [13, 14], the regional rate of health facility-based deliveries via a skilled birth attendant in former Western Province, where this study was conducted, are lower as compared to the national average (around 47 %) [14, 15].

### Research question

Given that over half of all deliveries are managed by TBAs in our catchment area, efforts by the Kenyan government to institute a community-based midwifery initiative, and the on-going debate within the global community regarding the most effective and least harmful manner by which to potentially integrate TBAs into the maternal newborn health (MNH) service continuum, we sought to ascertain the current practices of TBAs in former Western Province, Kenya (Bungoma, Busia, and Kakamega Counties). These descriptive data might help inform policy discussions regarding the roles TBAs could play in the maternal-newborn health landscape within Kenya, elsewhere in sub-Saharan Africa, and globally.

## Methods

The *Eunice Kennedy Shriver* National Institute of Child Health and Human Development (NICHD) Global Network for Women’s and Children’s Health Research (Global Network) was established in 2001 as a collaborative partnership of clinical researchers from South Asia, Africa, Latin America, and the United States. Global Network investigators conduct studies designed to test feasible and sustainable interventions to improve women’s and children’s health and to develop research capacity in low- and middle-income settings [16–25].

In 2010, seven of the Global Network sites participated in a survey of knowledge, attitudes and practices among Community Birth Attendants (CBAs), as described in Garces et al., 2012 [20]. In addition to the primary survey, our site in Western Kenya included an addendum of 11 questions in which issues specific to the practices of Kenyan community birth attendants were explored (Additional file 1). The results of the 11 question addendum are described in this paper.

Our catchment area is composed of 16 geographic clusters in Busia, Bungoma, and Kakamega Counties of Western Kenya. Cluster sizes vary from 25 to 65 km^2^, with 20–45 villages per cluster. An estimated 250–500 births occur annually per cluster. At the time the TBA practices survey was conducted, the Kenya Demographic and Health Survey (KDHS) of 2008–2009 indicated that nationally, 56 % of Kenyan births occurred at home, and 28 % of these were reported conducted by a TBA. The rates for home births conducted by TBAs in western Kenya, where this study was conducted, were considerably higher than the national average, at 73 % and 45 %, respectively [13].

Maternal and neonatal health research activities that are on-going or have occurred in our catchment area include a Maternal and Newborn Health Registry [17, 26], an EmONC implementation trial [27], nutrition studies [28], and *Helping Babies Breathe*^R^ a neonatal resuscitation training initiative [29, 30]. In addition, our study area abuts the catchment area of the USAID-AMPATH primary health care initiative [31–33].

One hundred TBAs from across the 16 Global Network clusters in Kenya were randomly selected by the Global Network data coordinating center (Research Triangle Institute, North Carolina) for face-to-face interviews that were conducted by a retired, bi-lingual Kenyan public health nurse who was specifically hired and trained to administer the survey in May-June 2010. Each TBA was interviewed individually, and the survey, including the Kenya-specific addendum, took about 1 h to complete. The survey was administered in English, the official language in Kenya, and was verbally translated into Kiswahili (national language), when necessary, by the interviewer. SPSS 19 [34] was used to perform descriptive analyses of the data.

This study was neither designed nor powered to perform cluster-level assessments; thus only overall descriptive results are reported. A total of 100 surveys were completed. On average, 6 TBAs were interviewed per cluster (range 4–7). One completed survey lacked information that would have identified the cluster to which the TBA belonged, but was inclusive of all other responses. Data from the survey with the missing cluster identification information were included in the final analyses.

## Results

### Supplies

The majority of TBAs (98 %) reported having access to gloves. Questions regarding access to other supplies and equipment are reported in the primary survey [20] and shown in Additional file 1.

### Management and referral practices for obstetric complications

All the methods reported to have ever been utilized to manage retained placental products among our sample of TBAs, and the frequency with which Kenyan TBAs reported using each method, are shown in Table 1.

**Table 1:** Reported methods to manage retained placental products among 100 Kenyan traditional birth attendants

Method	% TBAs report have ever used method^a^
Refer to health facility	61 %
Uterine massage	47 %
Manual extraction	15 %
Administer oxytocin	6 %
Other	16 %
Administer lantana leaves, herbs, or herbal oxytocin	9 %
Cooking stick or chain of beads down mother’s throat	3 %
Put beads down mother’s throat and rub a stone on the spine	1 %
Rub a grinding stone on mother’s back	1 %
Give mother hot porridge	1 %
Nipple massage	1 %

^a^TBAs were allowed to give more than one response; therefore, percentages do not total to 100 %

Most TBAs reported having ever referred women to health facilities for management by skilled personnel of obstetric emergencies such as obstructed labor (91 %), prolonged labor (88 %), bleeding (94 %), retained placenta (85 %), prolapsed cord (92 %), or seizures (82 %), while a lesser percentage reported referring pregnant women for hypertension (24 %). Fetal or newborn complications for which TBAs reported having ever made past health facility referrals of women and babies included: prematurity (81 %), breech presentation (71 %), no fetal heart rate (56 %), and baby not breathing after delivery (82 %). Other reported maternal and neonatal conditions which prompted referral by TBAs included “goiter,” “unable to push,” “big baby,” and “yellow baby,” although these were all infrequent reasons for referral, with only 1–2 % of TBAs reporting having ever referred because of these issues.

Nineteen percent of surveyed TBAs reported having ever used traditional substances for conditions related to obstructed or prolonged labor, retained placenta, maternal bleeding, fever, and lack of appetite (Table 1).

### Newborn care practices

The majority of TBAs surveyed (65 %) reported that they had never used mouth-to-mouth resuscitation to resuscitate a baby, 23 % said they’d previously used the technique, and 12 % of the surveys had this response missing. Twenty-three percent of respondents reported previously receiving *Helping Babies Breathe* neonatal resuscitation training, but most (98 %) had not received refresher training. Table 2 displays reported methods for umbilical cord care. Most TBAs reported applying “nothing” (44 %) or “spirit” (i.e., alcohol; 35 %) to the umbilicus, although 15 % also reported applying “powder.”

**Table 2:** Reported methods of umbilical cord care among 100 traditional birth attendants in western Kenya

Method	% TBAs report currently using method^a^
Do Nothing	44 %
Apply spirit (isopropyl alcohol)	35 %
Apply Powder	15 %
Apply Oil	2 %
Apply Powder and oil	1 %
Apply Jik (bleach)	1 %
Missing response	2 %
Apply Gentian violet	0 %

^a^TBAs allowed to choose only one response

### Reporting and Remuneration practices

The majority of TBAs (84 %) report pregnancy and delivery outcomes to their chief and/or village elder.

**Fig. 1:**
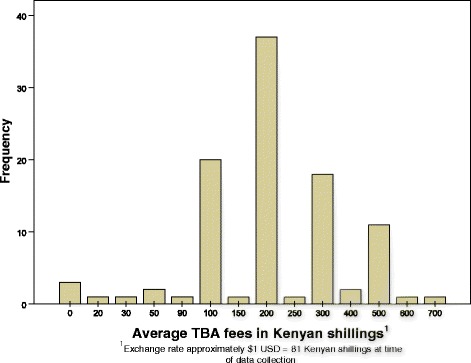
Kenya TBA self-reported Fees for delivery services

Birth attendants were asked if they charged to provide services to women in their community; 97 % answered in the affirmative. The reported range of charges for delivering with a TBA in our sample was 20–700 Kenyan Shillings (KES; about $0.25–$8.64, USD, using an exchange rate at the time of the survey of 81 KES:$1 USD), with 79 % of these TBAs reporting that they charge between 100 and 300 KES ($1.23–$3.70 USD; Fig. 1). The majority of TBAs (80 %) would not accept barter for their services, and exclusively required monetary compensation. Of those TBAs who did accept barter, food items (e.g., chicken, maize, cassava, sugar, tea leaves), rather than non-food items or an exchange of services, were exclusively accepted.

### Perceived relationship with local health facilities

**Fig. 2:**
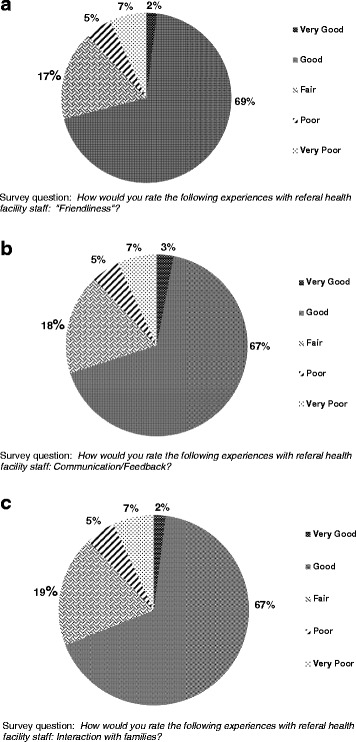
**a**: Kenyan TBA ratings for “*Friendliness of health facility staff*”. **b**: Kenyan TBA ratings for “*Communication with health facility staff*”. **c**: Kenyan TBA ratings for “*How families interact with health facility staff*”

Using a 5-point scale ranging from “Very Poor” to “Very Good,” TBAs generally rated their local health facilities as “Good” to “Fair” on friendliness (“Good” = 69 %; “Fair” = 17 %; Fig. 2a), communication (68 %; 18 %; Fig. 2b), and interaction with families (68 %; 19 %; Fig. 2c).

## Discussion

The most worrisome finding of this descriptive study is that, during the survey period, among 100 TBAs sampled in Western Kenya, some self-report the use of maternal and newborn care practices that are, in some cases, outdated (e.g., applying substances to the umbilical cord stump), and in other instances, potentially harmful (e.g., putting a cooking stick down a mother’s throat to manage retained placental products). Encouraging results from this survey indicate that: (1) most TBAs report that they have access to gloves; (2) a high proportion of TBAs in our sample self-report that the perceived relationship with their local health facility is “good” or “fair;” (3) a high percentage of TBAs report having ever referred pregnant women to health facilities for advanced care of common obstetric complications; and (4) the majority of TBAs report pregnancy and birth outcomes to their village elders and chiefs.

Some TBAs in our limited sample report troubling gaps in maternal and neonatal care knowledge, and potentially harmful maternal and newborn care practices. These results align with those found in similar settings [35]. In the current study, we did not investigate how these gaps in knowledge and practice might directly or indirectly impact maternal or newborn morbidity and mortality in our catchment area. However, a recent report from our region, based on data from the Health and Demographic Surveillance System, found significantly higher rates of mortality among women in lower socio-economic groups who sought care from TBAs [36].

There are some important study limitations to consider: One is that the findings are based on self-report. TBAs were interviewed as to current practices and attitudes; additional observations were not conducted to confirm the veracity of TBA self-reporting. Another key limitation is that this was a descriptive study of a small sample of traditional birth attendants working in a defined catchment area in western Kenya. As such, our results might not be wholly representative of TBAs in other parts of Kenya or other global regions.

It is also possible that our survey results actually *overestimate* the abilities of Kenyan TBAs, and the positive relationships that they report with local health facilities. Although the interviewer was a retired public health nurse, the TBA survey respondents may have known, or have worked with her in the past, thereby positively biasing their responses. In addition, the survey was conducted among a small sample of TBAs in western Kenya, an area heavily penetrated by various maternal-newborn clinical and research initiatives [21–24, 33, 37–41].

Thus, TBAs in our sample may have greater access to training and educational opportunities than TBAs in other regions of Kenya, as well as better linkage with staff at their local health facilities. This may have resulted in a higher baseline-level of education, training, and cross-pollination of shared knowledge and skills than is typical of TBA populations in other settings.

Despite these limitations, we believe the data reported add important information to the current state of knowledge regarding the manner by which TBAs intersect with their communities, including with pregnant women and local health facilities. Our combined findings may be particularly important within the current global context of an on-going conversation regarding the most effective manner by which to reduce maternal and newborn mortality, and in light of recent findings from the 2014 Kenya Demographic Health Survey, which demonstrate an overall reduction in rates in infant, child, and under-5 mortality [14]; these reductions may, or may not, be related, in part, to Government of Kenya and partner efforts to implement community midwifery initiatives.

Community midwifery is a highly desirable and culturally acceptable option for pregnant Kenyan women and the communities within which they live [12]; for many regional locations where the burden of maternal and neonatal mortality and morbidity are highest, and Emergency Obstetric and Neonatal Care (EmONC) strategies are rolling out, TBAs and other community-based health worker cadres continue to be a strong and influential force in the lives of pregnant women and their families [15, 36, 42, 43]. Given the influence of this existing cadre of birth attendants on women’s attitudes toward pregnancy, birth, and newborn care, it seems community-level birth attendants could potentially have their roles as care providers redefined to that of: (a) valued educators (e.g., regarding the importance of immunization, appropriate cord care, early/exclusive breastfeeding [25], and hygiene); (b) as advocates for skilled, facility-based delivery; (c) as birth companions who offer psychosocial support for pregnant and delivering mothers; and (d) in some instances, such as during neonatal resuscitation and essential newborn care, as a semi-skilled “extra pair of hands” to assist more highly trained—but often understaffed and overburdened--health workers [2, 5, 44–50]. On a practical level, this may imply that “re-branding” of TBAs as birth companions, labor coaches, and semi-skilled helpers who accompany laboring women to EmONC-equipped facilities for delivery, is a potential model by which to increase access to skilled birth attendance for women living in LMICs.

Within this context, data from the current study may be of particular interest for policymakers in sub-Saharan Africa and other global regions where there is on-going discussion regarding the most effective roles and responsibilities of TBAs within the maternal-newborn-child health care continuum [51]. For example, results from our survey indicate that remuneration may be a potential incentive by which to successfully motivate task-shifting and/or re-branding among community-based birth attendants. Furthermore, because prior investigations have shown that the most successful TBA initiatives are those which link educated TBAs and pregnant women with skilled health care providers for on-going support and supervision, and to EmONC-equipped facilities for referral [52–56], careful consideration should be directed toward how to most effectively link, train, and equip community based birth companions within the existing health infrastructure so as to maximize positive impact and minimize potential harm to mothers and babies.

## Conclusions

Within the limitations of a small sample size and restricted geographical location within Kenya, results from our descriptive study indicate both encouraging results regarding the self-reported willingness of TBAs to link pregnant women with local health facilities for provision of MNH services and potentially troubling gaps in key MNH knowledge and practices among TBAs. In order to address these findings, stakeholders and policy-makers from LMICs may want to consider whether it might be suitable to employ strategies of task-shifting, role redefinition, and re-education of traditional birth attendants, especially in regards to high-impact, low-cost MNH interventions such as hand hygiene, cord care, timely initiation of exclusive breastfeeding, prevention of hypothermia, and basic neonatal resuscitation.

## Abbreviations

EmONC, emergency obstetric and newborn care; KDHS, Kenya demographic and health survey; KES, Kenyan shilling; LMICs, low- and middle-income countries; MNH, maternal newborn health; TBA, traditional birth attendant

## Additional files

Additional file 1: The Global Network Community Birth Attendant Survey tool used during the current study (Kenya site specific questions, pp. 14–15) and as reported in Garces et al. (2012) [20]. (DOC 392 kb) Additional file 2: Frequency tables for self-reported practices of TBAs in Western Kenya from which the results and findings for the descriptive study reported in this manuscript are derived. (PDF 40 kb)

## References

[1] Bukar M, Jauro YS (2013). Home births and postnatal practices in Madagali, north-eastern Nigeria. Niger J Clin Pract.

[2] Prata N, Quaiyum MA, Passano P, Bell S, Bohl DD, Hossain S, Azmi AJ, Begum M (2012). Training traditional birth attendants to use misoprostol and an absorbent delivery mat in home births. Soc. Sci. Med.

[3] Montagu D, Yamey G, Visconti A, Harding A, Yoong J (2011). Where do poor women in developing countries give birth? A multi-country analysis of demographic and health survey data. PLoS One.

[4] Engmann C (2011). Improving neonatal mortality in sub-Saharan Africa: any cause for optimism?. J. Perinatol.

[5] Prata N, Passano P, Rowen T, Bell S, Walsh J, Potts M (2011). Where there are (few) skilled birth attendants. J Health Popul Nutr.

[6] Yakoob MY, Ali MA, Ali MU, Imdad A, Lawn JE, Van Den Broek N, Bhutta ZA (2011). The effect of providing skilled birth attendance and emergency obstetric care in preventing stillbirths. BMC Public Health.

[7] Bellows B, Kyobutungi C, Mutua MK, Warren C, Ezeh A. Increase in facility-based deliveries associated with a maternal health voucher programme in informal settlements in Nairobi, Kenya. Health Policy Plan. 2013;28(2):134–42 doi: 10.1093/heapol/czs030. 10.1093/heapol/czs030 PMC3584990 22437506

[8] Sidney K, Decosta A, Diwan V, Mavalankar DV, Smith H (2012). An evaluation of two large scale demand side financing programs for maternal health in India: the MATIND study protocol. BMC Public Health.

[9] Whitaker K, Blog PM (2012). Is Sierra Leone right to ban traditional birth attendants?. Guardian.

[10] Murigi S, Ford L. Should Uganda ban traditional birth attendants? In: Katine Chronicles Blog. London, UK: Guardian News and Media Limited; 2010.

[11] Mariita A (2011). Shold Kenya still use traditional birth attendants?. The Key correspondents.

[12] Mannah MT, Warren C, Kuria S, Adegoke AA (2014). Opportunities and challenges in implementing community based skilled birth attendance strategy in Kenya. BMC Pregnancy Childbirth.

[13] KNBS (2010). Kenya demographic and health survey 2008–09.

[14] KNBS, MOH, NACC, KEMRI, NCPD. 2014 Kenya Demographic and Health Survey: Key indicators. In. Nairobi, Kenya; 2015.

[15] Council P (2003). Traditional birth attendants in maternal health programmes. safeMOTHERhood.

[16] Carlo W, Goudar S, Jehan I, Chomba E, Tshefu A, Garces A (2010). Newborn care training and perinatal mortality in communities in developing countries. N Engl J Med.

[17] Goudar S, Carlo W, McClure E (2012). The maternal and newborn health registry study of the global network for Women’s and Children’s health research. Int J Gynaecol Obstet.

[18] Krebs N, Hambidge K, Mazariegos M, Westcott J, Goco N, Wright L (2011). Complementary feeding: a global network cluster randomized controlled trial. BMC Pediatr.

[19] Bucher S, Marete I, Tenge C, Liechty EA, Esamai F, Patel A, Goudar SS, Kodkany B, Garces A, Chomba E, Althabe F, Barreuta M, Pasha O, Hibberd P, Derman RJ, Otieno K, Hambidge K, Krebs NF, Carlo WA, Chemweno C, Goldenberg RL, McClure EM, Moore JL, Wallace DD, Saleem S, Koso-Thomas M A prospective observational description of frequency and timing of antenatal care attendance and coverage of selected interventions from sites in Argentina, Guatemala, India, Kenya, Pakistan, and Zambia Reprod Health. 2015;12 Suppl 2:S12. doi:10.1186/1742-4755-12-S2-S12. Epub 2015 Jun 8 10.1186/1742-4755-12-S2-S12 PMC4464209 26063483

[20] Garces A, McClure E, Chomba E, Patel A, Pasha O, Tshefu A, Esamai F, Goudar SS, Lokangaka A, Hambidge KM (2012). Home birth attendants in low income countries: who are they and what do they do?. BMC Pregnancy Childbirth.

[21] Gisore P, Rono B, Marete I, Nekesa-Mangeni J, Tenge C, Shipala E, Mabeya H, Odhiambo D, Otieno K, Bucher S (2013). Commonly cited incentives in the community implementation of the emergency maternal and newborn care study in western Kenya. Afr Health Sci.

[22] Gisore P, Shipala E, Otieno K, Rono B, Marete I, Tenge C, Mabeya H, Bucher S, Moore J, Liechty E (2012). Community based weighing of newborns and use of mobile phones by village elders in rural settings in Kenya: a decentralised approach to health care provision. BMC Pregnancy Childbirth.

[23] Liechty E, Esamai F, McClure E, Moore J, Bucher S, Tenge C, Marete I, Koso-Thomas M, Wright L (2011). Mobile phones to engage village elders in case finding for a birth registry in rural Kenya.

[24] Marete I, Tenge C, Chemweno C, Bucher S, Pasha O, Ramadurg UY, Mastiholi SC, Chiwila M, Patel A, Althabe F, Garces A, Moore JL, Liechty EA, Derman RJ, Hibberd PL, Hambidge K, Goldenberg RL, Carlo WA, Koso-Thomas M, McClure EM, Esamai F. Loss to followup among pregnant women in a multi-country, community-based maternal and newborn health registry: A prospective, cohort study. Reprod Health. 2015;12 Suppl 2:S4. doi: 10.1186/1742-4755-12-S2-S4. Epub 2015 Jun 8. 10.1186/1742-4755-12-S2-S4 PMC4464022 26062899

[25] Patel A, Bucher S, Pusdekar Y, Esamai F, Krebs N, Goudar S, Chomba E, Garces A, Pasha O, Saleem S (2015). Rates and determinants of early initiation of breastfeeding and exclusive breast feeding at 42 days postnatal in six low and middle-income countries: a prospective cohort study. Reprod Health.

[26] Bose C, Bauserman M, Goldenberg R, Goudar S, McClure E, Pasha O (2015). The global network maternal newborn health registry: a multi-national, community-based registry of pregnancy outcomes. Reprod. Health.

[27] Pasha O, Goldenberg RL, McClure EM, Saleem S, Goudar SS, Althabe F, Patel A, Esamai F, Garces A, Chomba E (2010). Communities, birth attendants and health facilities: a continuum of emergency maternal and newborn care (the global Network’s EmONC trial). BMC Pregnancy Childbirth.

[28] Esamai F, Liechty E, Ikemeri J, Westcott J, Kemp J, Culbertson D, Miller LV, Hambidge KM, Krebs NF (2014). Zinc absorption from micronutrient powder is low but is not affected by iron in Kenyan infants. Nutrients.

[29] Singhal N, Lockyer J, Fidler H, Keenan W, Little G, Bucher S (2012). Helping Babies Breathe: global neonatal resuscitation program development and formative educational evaluation. Resuscitation.

[30] Bang A, Bellad R, Gisore P, Hibberd P, Patel A, Goudar S (2014). Implementation and evaluation of the helping babies breathe curriculum in three resource limited settings: does helping babies breathe save lives? A study protocol. BMC Pregnancy Childbirth.

[31] Braitstein P, Siika A, Hogan J, Kosgei R, Sang E, Sidle J, Wools-Kaloustian K, Keter A, Mamlin J, Kimaiyo S (2012). A clinician-nurse model to reduce early mortality and increase clinic retention among high-risk HIV-infected patients initiating combination antiretroviral treatment. J Int AIDS Soc.

[32] Nyandiko WM, Otieno-Nyunya B, Musick B, Bucher-Yiannoutsos S, Akhaabi P, Lane K, Yiannoutsos CT, Wools-Kaloustian K (2010). Cost-effectiveness of first-line antiretroviral therapy for HIV-infected African children less than 3 years of age. J Acquir Immune Defic Syndr.

[33] Wools-Kaloustian K, Eller A, Otieno-Nyunya B, Akhaabi P, Puri R, Bucher S, Nyandiko W (2012). Traditional birth attendants: a forgotten resource in HIV education and prevention. Obstetrics Gynaecol Eastern Central Africa.

[34] IBM (2010). IBM SPSS statistics for windows. Version 190.

[35] Beltman JJ, van den Akker T, Bwirire D, Korevaar A, Chidakwani R, van Lonkhuijzen L, van Roosmalen J (2013). Local health workers’ perceptions of substandard care in the management of obstetric hemorrhage in rural Malawi. BMC Pregnancy Childbirth.

[36] Desai M, Phillips-Howard PA, Odhiambo FO, Katana A, Ouma P, Hamel MJ, Omoto J, Macharia S, van Eijk A, Ogwang S (2013). An analysis of pregnancy-related mortality in the KEMRI/CDC health and demographic surveillance system in western Kenya. PLoS One.

[37] Bucher S, Marete I, Tenge C, Liechty E, Esamai F, Patel A, Goudar S, Kodkany B, Garces A, Chomba E (2015). A prospective observational description of frequency and timing of antenatal care attendance and coverage of selected interventions from sites in Argentina, Guatemala, India, Kenya, Pakistan and Zambia. Reprod. Health.

[38] Eslami P, Bucher S, Mungai R (2015). Improper reprocessing of neonatal resuscitation equipment in rural Kenya compromises function: recommendations for more effective implementation of helping babies breathe. Resuscitation.

[39] Thukral A, Lockyer J, Bucher SL, Berkelhamer S, Bose C, Deorari A, Esamai F, Faremo S, Keenan WJ, McMillan D (2015). Evaluation of an educational program for essential newborn care in resource-limited settings: essential care for every baby. BMC Pediatr.

[40] ASH, Bucher SL. mHBB: Using mobile phones to support Helping Babies Breathe in Kenya. In: mHealth Compendium volume 5. Edited by USAID. Arlington, Virginia; 2015. p. 46–47.

[41] Esamai F, Tshefu A, Ayede A, Adejuyigbe E, Wammanda R, Baqui A (2013). Ongoing trials of simplified antibiotic regimens for the treatment of serious infections in young infants in South Asia and sub-Saharan Africa: implications for policy. Pediatr Infect Dis J.

[42] Oyerinde K, Harding Y, Amara P, Garbrah-Aidoo N, Kanu R, Oulare M, Shoo R, Daoh K. A Qualitative Evaluation of the Choice of Traditional Birth Attendants for Maternity Care in 2008 Sierra Leone: Implications for Universal Skilled Attendance at Delivery. Matern Child Health J. 2013;17(5):862–8. doi: 10.1007/s10995-012-1061-4. 10.1007/s10995-012-1061-4 22736032

[43] Wilunda C, Quaglio G, Putoto G, Lochoro P, Dall’Oglio G, Manenti F, Atzori A, Lochiam RM, Takahashi R, Mukundwa A (2014). A qualitative study on barriers to utilisation of institutional delivery services in Moroto and Napak districts, Uganda: implications for programming. BMC Pregnancy Childbirth.

[44] Byrne A, Morgan A (2011). How the integration of traditional birth attendants with formal health systems can increase skilled birth attendance. Int J Gynaecol Obstet.

[45] Okonofua F, Ogu R (2014). Traditional versus birth attendants in provision of maternity care: call for paradigm shift. Afr J Reprod Health.

[46] Mobeen N, Durocher J, Zuberi N, Jahan N, Blum J, Wasim S, Walraven G, Hatcher J (2011). Administration of misoprostol by trained traditional birth attendants to prevent postpartum haemorrhage in homebirths in Pakistan: a randomised placebo-controlled trial. BJOG.

[47] Prata N, Ejembi C, Fraser A, Shittu O, Minkler M (2012). Community mobilization to reduce postpartum hemorrhage in home births in northern Nigeria. Soc. Sci. Med.

[48] Prata N, Passano P, Bell S, Rowen T, Potts M. New hope: community-based misoprostol use to prevent postpartum haemorrhage. Health Policy Plan. 2013;28(4):339–46. doi: 10.1093/heapol/czs068. Epub 2012 Aug 9 10.1093/heapol/czs068 22879523

[49] Rowen T, Prata N, Passano P (2011). Evaluation of a traditional birth attendant training programme in Bangladesh. Midwifery.

[50] Mduma E, Ersdal H, Svensen E, Kidanto H, Auestad B, Perlman J (2015). Frequent brief on-site simulation training and reduction in 24-h neonatal mortality—an educational intervention study. Resuscitation.

[51] Colvin CJ, de Heer J, Winterton L, Mellenkamp M, Glenton C, Noyes J, Lewin S, Rashidian A (2013). A systematic review of qualitative evidence on barriers and facilitators to the implementation of task-shifting in midwifery services. Midwifery.

[52] Bhutta ZA, Lassi ZS, Blanc A, Donnay F (2010). Linkages among reproductive health, maternal health, and perinatal outcomes. Semin Perinatol.

[53] Barros FC, Bhutta ZA, Batra M, Hansen TN, Victora CG, Rubens CE (2010). Global report on preterm birth and stillbirth (3 of 7): evidence for effectiveness of interventions. BMC Pregnancy Childbirth.

[54] Bhutta ZA, Yakoob MY, Lawn JE, Rizvi A, Friberg IK, Weissman E, Buchmann E, Goldenberg RL (2011). Stillbirths: what difference can we make and at what cost?. Lancet.

[55] Ariff S, Soofi SB, Sadiq K, Feroze AB, Khan S, Jafarey SN, Ali N, Bhutta ZA (2010). Evaluation of health workforce competence in maternal and neonatal issues in public health sector of Pakistan: an assessment of their training needs. BMC Health Serv Res.

[56] Bhopal SS, Halpin SJ, Gerein N (2013). Emergency obstetric referral in rural Sierra Leone: what can motorbike ambulances contribute? a mixed-methods study. Matern Child Health J.

