# Supplementary material for: Self-reported practices among traditional birth attendants surveyed in western Kenya: a descriptive study
Source: BMC Pregnancy Childbirth. 2016 Aug 12;16:219. doi: 10.1186/s12884-016-1007-8 (PMC4981994; doi:10.1186/s12884-016-1007-8)
Supplement: Additional file 1: — The Global Network Community Birth Attendant Survey tool used during the current study (Kenya site specific questions, pp. 14–15) and as reported in Garces et al. (2012) [20]. (DOC 392 kb) [file 12884_2016_1007_MOESM1_ESM.doc]

**GLOBAL NETWORK: Community Birth Attendants Survey**

**Date of Interview**: |__|__| / |__|__| / |__|__|__|__| **Cluster**: __________________

DayMonthYear

**Birth Attendant Name**: ____________________________**Interviewer Name**: _____________________________

**Birth Attendant ID**: |__|__|__|__|

**Birth Attendant Address**: _____________________________________________________________________________

**Introduction and Verbal Consent**

**MOI UNIVERSITY**

**A Survey of Community Birth Attendants’ Knowledge, Practices and Role within the Health Care System**

***INFORMED CONSENT FORM: Birth Attendants***

**Sponsors**

This study is being conducted by the Global Network for Women’s and Children’s Health Research, in 6 countries. It is funded by the U.S. National Institutes of Health

**Purpose of the Study**

The Global Network is doing a research study of birth attendants´ knowledge and reported practices relating to the care that is given to mothers and babies in communities in developing countries. The purpose is to better understand how you deliver babies and practices you use to keep mothers and babies safe during and following birth.

**What will be done in the study?**

We will ask you questions about your training and how you practice. We will include your answers, which will be anonymous (secret), in the evaluation of this study. There is no cost for participating in the survey. There will be no payment for participants.

**Who do I contact if I have questions?**

If you have questions about this study, you should contact Prof. Fabian Esamai on Tel 254733836410 or 254724400189. If you have questions about your rights, please contact Prof. David Ngare the Chairman of the Institutional Reasearch and Ethics Committee (IREC) of MTRH/MU School of Medicine.

If you decide not to participate in the study, this will not influence your activities. You can withdraw from the survey or choose not to answer a specific question at any time.

We have given you information about the project called “The Global Network: Community Birth Attendant Survey”. You do not have to agree to be in the survey or may decide later not to be part of the project. This will not affect your training or employment in any way. If you have any questions, please call [insert senior investigator/ethics committee].

***If you agree with the statement above:***

Printed Name of Study Participant --------------------------------------------- Date: ---------------------------

Signature of Study Participant ------------------------------------------------

Printed Name of Person Obtaining Consent --------------------------------- Date: ---------------------------

Signature of Person Obtaining Consent ------------------------------------

**For subjects who cannot read and write, a witness must sign here:**

**I was present while the benefits, risk and procedures were read to the participant. The participant had an opportunity to ask questions she might have about the study and agreed to take part in this research.**

Printed Name of Witness ______________________________________ Date: _____________

Signature of Witness

**Section A. Respondent Characteristics**

| **First, I have some questions about you.**  **A01**.How old are you? (IF UNKNOWN SAY: Your best estimate is fine.)  ___ ___ YEARS OLD (range 12 – 90 years)  **A02**. CODE R SEX (IF UNSURE, ASK: Are you female or male?)  1 FEMALE 2 MALE  **A03**. Is ________ your primary language? Can you speak it? Can you write it? What about reading it? (MARK ALL THAT APPLY)   |  |  | Not Applicable | Primary Language | Can Speak | Can  Write | Can  Read | | --- | --- | --- | --- | --- | --- | --- | | **a.** | English? | 0 | 1 | 2 | 3 | 4 | | **b.** | Spanish? | 0 | 1 | 2 | 3 | 4 | | **c.** | Cahiquel? | 0 | 1 | 2 | 3 | 4 | | **d.** | Urdu? | 0 | 1 | 2 | 3 | 4 | | **e.** | Ngbaka? | 0 | 1 | 2 | 3 | 4 | | **f.** | Swahili? | 0 | 1 | 2 | 3 | 4 | | **g.** | French? | 0 | 1 | 2 | 3 | 4 | | **h.** | ________ | 0 | 1 | 2 | 3 | 4 |   **A04**. How many years of formal schooling have you completed?  ___ ___ YEARS (ENTER “0” IF NO FORMAL SCHOOLING) (range 0 – 30 years)  **A05**. How many years have you been delivering babies?  ___ ___ YEARS (range 0 – 80 years) | | **A06**. I am going to read a list of activities. For each one, please tell me if it applies to you.   |  |  | **Yes** | **No** | | --- | --- | --- | --- | | **a.** | I can read numbers. | 1 | 2 | | **b.** | I can write numbers. | 1 | 2 | | **c.** | I can use a calendar. | 1 | 2 | | **d.** | I can tell time. | 1 | 2 | | **e.** | I own a cell phone. | 1 | 2 | | **f.** | I cook over an open fire. | 1 | 2 | | **g.** | I have access to a bike. | 1 | 2 | | **h.** | I have access to a car, truck, or motorbike. | 1 | 2 | | **i.** | My household has a working television. | 1 | 2 | | **j.** | My household has a working radio. | 1 | 2 | | **k.** | My household has an indoor toilet. | 1 | 2 | | **l.** | My household has electricity. | 1 | 2 |   **A07**. Do you have a watch?  1 YES  2 NO 🡪 **GO TO SECTION B ON PAGE 3**  **A08**. Do you carry it with you to deliveries?  1 YES  2 NO |
| --- | --- | --- | --- | --- | --- | --- | --- | --- | --- | --- | --- | --- | --- | --- | --- | --- | --- | --- | --- | --- | --- | --- | --- | --- | --- | --- | --- | --- | --- | --- | --- | --- | --- | --- | --- | --- | --- | --- | --- | --- | --- | --- | --- | --- | --- | --- | --- | --- | --- | --- | --- | --- | --- | --- | --- | --- | --- | --- | --- | --- | --- | --- | --- | --- | --- | --- | --- | --- | --- | --- | --- | --- | --- | --- | --- | --- | --- | --- | --- | --- | --- | --- | --- | --- | --- | --- | --- | --- | --- | --- | --- | --- | --- | --- | --- | --- | --- | --- | --- | --- | --- | --- | --- | --- | --- | --- | --- | --- | --- | --- | --- | --- | --- | --- | --- | --- | --- |
|  |  | |

**Section B. Birth Attendant Training**

**B01**. Have you ever undergone formal schooling or training lasting **one month or more**? Do not include training from another CBA or community member.

1 YES

2 NO 🡪 **GO TO QUESTION B02 ON PAGE 4**

**Table B01. Long Term Training or Schooling**

COMPLETE A SEPARATE ROW FOR EACH TRAINING/COURSE THAT WAS 4 WEEKS OR MORE IN LENGTH (ADD ROWS AS NEEDED).

| **B01** | **a. Organization** | **b. Subject or Topic**  *Record all the apply* | **c. Date (Year)** | **d. Length of training [in weeks]** | **e. Was your tuition free?** | **f. Was a degree obtained?** | **g. (IF YES) List degree received.** |
| --- | --- | --- | --- | --- | --- | --- | --- |
| CODES: | 1 = Public University 2 = Private University 3 = Other institution 4 = Other  5 = DK | 1 = General health 2 = Prenatal care 3 = Newborn care 4 = Maternal care 5 = Family Planning 6 = Other | 20___ ___  (Enter 9999 if DK) | this column hill have 2 boxes | 1 = Yes 2 = No 3 = DK | 1 = Yes 2 = No 3 = DK |  |
| **1.** |  |  |  |  |  |  |  |
| **2.** |  |  |  |  |  |  |  |
| **3.** |  |  |  |  |  |  |  |
| **4.** |  |  |  |  |  |  |  |
| **5.** |  |  |  |  |  |  |  |
| **6.** |  |  |  |  |  |  |  |
| **7.** |  |  |  |  |  |  |  |

**B02**. Have you received training of **less than one month** in length in the last 5 years?

1 YES

2 NO 🡪 **GO TO SECTION C ON PAGE 5**

**Table B02. Short-Term Training or Course**

ENTER A SEPARATE ROW FOR EACH TRAINING RECEIVED IN LAST 5 YEARS (ADD ROWS AS NEEDED).

| **B02** | **a. Organization which provided training course** | **b. Subject or Topic** *Record all the apply* | **c. Date (Year)**  *If the same training is given each year, list all years.* | **d. Length of training**  *If training*  *more than 4 weeks, enter in Table B1* | **e. Was a certificate given?** | **f. Was follow-up or refresher training given after the initial training?** | **g. Were you paid to attend?** |
| --- | --- | --- | --- | --- | --- | --- | --- |
| CODES: | 1 = MOH or clinic or   government 2 = NGO 3 = Private institution 4 = Other 5 = DK | 1 = vaccination 2 = prenatal care 3 = newborn care 4 = maternal care 5 = Family Planning 6 = DK/DR | 20___ ___  (Enter 9999 if DK) | 1 = < 1 day  2 = 1 day 3 = 2 to 6 days 4 = 1 to 2 weeks 5 = 2 to 4 weeks | 1 = Yes 2 = No  3 = DK | 1 = Yes 2 = No  3 = DK | 1 = Yes 2 = No  3 = DK |
| **1.** |  |  |  |  |  |  |  |
| **2.** |  |  |  |  |  |  |  |
| **3.** |  |  |  |  |  |  |  |
| **4.** |  |  |  |  |  |  |  |
| **5.** |  |  |  |  |  |  |  |
| **6.** |  |  |  |  |  |  |  |
| **7.** |  |  |  |  |  |  |  |

**Section C. Birth Attendant Practices**

| **Now, let’s talk more about your work as a birth attendant.**  **C01.** Thinking about the number of births you attend each month, would you say you attended… (PLEASE INCLUDE ALL BIRTHS, NO MATTER WHAT THE OUTCOME)  1 None or less than one?  2 One to two?  3 Three to four?  4 Five or more?  **C02.** Where do you attend births? (MARK ALL THAT APPLY.)  **a** CBA’s house  **b** Mother’s house  **c** Health facility  **d** Other 🡪 Specify: **e**.________________________________ | **C03.** For the average woman who you care for, how many prenatal visits do you routinely carry out?  1 NONE 🡪 **GO TO QUESTION C05**  2 1 to 2  3 3 to 4  4 5 to 6  5 7 to 8  6 9 OR MORE  **C04.** During prenatal visits, how often do you advise the mother to visit a health facility for the following problems?   |  |  | **Never** | **Sometimes** | **Always** | | --- | --- | --- | --- | --- | | **a.** | Vaginal bleeding | 1 | 2 | 3 | | **b.** | Severe swelling | 1 | 2 | 3 | | **c.** | Fever | 1 | 2 | 3 | | **d.** | Other:**e**.___________ | 1 | 2 | 3 | |
| --- | --- | --- | --- | --- | --- | --- | --- | --- | --- | --- | --- | --- | --- | --- | --- | --- | --- | --- | --- | --- | --- | --- | --- | --- | --- | --- |

**INTERVIEWER**: For this section you should show the CBA the equipment to assess whether they are familiar with how to use it. If CBA is unable to correctly demonstrate use of the equipment, mark in the interviewer confirmation box.

| **Maternal Weight** | | **Yes** | **No** |  |  | **Yes** | **No** |  | **Interviewer Confirmation** |
| --- | --- | --- | --- | --- | --- | --- | --- | --- | --- |
| **C05.** | Do you have access to a scale to weigh mothers? | 1 | 2 |  | **C06.** (IF YES) Can you get the weight of the mother? | 1 | 2 |  | **C07**. 0 R unable |
| **C08.** | Do you refer mothers for low or high weight? | 1 | 2 |  |  |  |  |  |  |

| **Blood Pressure** | | **Yes** | **No** |  |  | **Yes** | **No** |  | **Interviewer Confirmation** |
| --- | --- | --- | --- | --- | --- | --- | --- | --- | --- |
| **C09.** | Do you have a stethoscope? | 1 | 2 |  | **C10.** (IF YES) Can you count the heart beat? | 1 | 2 |  | **C11.** 0 R unable |
|  |  |  |  |  | **C10a.** Do you ever use it to listen to fetal heartbeat? | 1 | 2 |  |  |
| **C12.** | Do you have a blood pressure cuff? | 1 | 2 |  | **C13.** (IF YES) Can you read the cuff? | 1 | 2 |  | **C14.** 0 R unable |
| **C15.** | Do you generally take blood pressure? | 1 | 2 |  | **C16.** (IF YES) Do you know what the normal range of blood pressure is? | 1 | 2 |  | **C17.** 0 R unable |
|  |  |  |  |  | **C16a.** Do you take blood pressures prenatally? | 1 | 2 |  |  |
|  |  |  |  |  | **C16b.** Do you take blood pressures during labor? | 1 | 2 |  |  |

| **Other Tests** | | **Yes** | **No** |  |  | **Yes** | **No** |  | **Interviewer Confirmation** |
| --- | --- | --- | --- | --- | --- | --- | --- | --- | --- |
| **C18.** | Do you have dipsticks to test for urine protein? | 1 | 2 |  | **C19.** (IF YES) Do you administer this test? | 1 | 2 |  |  |
|  |  |  |  |  | **C19a.** Can you tell if the test is positive? | 1 | 2 |  | **C20.** 0 R unable |
| **C21.** | Do you have dipsticks to test for sugar in urine? | 1 | 2 |  | **C22.** (IF YES) Do you administer this test? | 1 | 2 |  |  |
|  |  |  |  |  | **C22a.** Can you tell if the test is positive? | 1 | 2 |  | **C23.** 0 R unable |
| **C24.** | Do you have dipsticks to test for bacteria in urine? | 1 | 2 |  | **C25.** (IF YES) Do you administer this test? | 1 | 2 |  |  |
|  |  |  |  |  | **C25a.** Can you tell if the test is positive? | 1 | 2 |  | **C26.** 0 R unable |
| **C27.** | Do you have a test for maternal hemoglobin? | 1 | 2 |  | **C28.** (IF YES) What type of test do you have? __________________________ |  |  |  |  |
|  |  |  |  |  | **C28a.** Do you administer this test? | 1 | 2 |  |  |
|  |  |  |  |  | **C28b.** Do you know how to interpret this test? | 1 | 2 |  | **C29.** 0 R unable |
| **C30.** | Do you have a fetoscope? | 1 | 2 |  | **C31.** (IF YES) Do you use it to obtain fetal heart beat? | 1 | 2 |  |  |

| **C32.** Which of the following tests have you ever sent to a laboratory?  (MARK ALL THE APPLY)  a Urine test for protein?  b Urine test for bacteria?  c Urine test for sugar?  d Fecal tests for parasites?  e Blood test for anemia?  f  HIV test?  g Hepatitis test?  h Syphilis test?  i  Malaria?  j  Other? Specify k. _______________________  k None?  **C33.** Now, I have some questions about using a thermometer and a tape measure.   |  |  | **Yes** | **No** | | --- | --- | --- | --- | | **a.** | Do you ever use a thermometer to diagnose fever? | 1 | 2 | | **b.** | Do you have a thermometer? | 1 | 2 | | **c.** | Can you read the scale on a thermometer? | 1 | 2 | | **d.** | Do you ever use a tape to measure the height of the fundus? | 1 | 2 | | **e.** | Can you read numbers on the tape? | 1 | 2 |   **C34.** Can you estimate when the baby is due…   |  |  | **Yes** | **No** | | --- | --- | --- | --- | | **a.** | by the mother’s last menstrual period? | 1 | 2 | | **b.** | by the top of the fundus? | 1 | 2 | | **c.** | by the estimated size? | 1 | 2 | | **d.** | by another method? | 1 | 2 | | **C35.** When the woman is in labor, do you ever try to determine if the baby will be premature (too small)?  1 YES  2 NO  **C36.** Do you know if the mother has pre-eclampsia or is likely to have a seizure by checking…   |  |  | **Yes** | **No** | | --- | --- | --- | --- | | **a.** | BP? | 1 | 2 | | **b.** | protein in the urine? | 1 | 2 | | **c.** | the amount of swelling? | 1 | 2 |   **C37.** During prenatal visits, do you give advice regarding a danger sign or danger signs requiring a visit to a health facility?  1 YES  2 NO 🡪 **GO TO QUESTION C39**  **C38.** During prenatal visits, would you say that you never, sometimes, or always give advice regarding the danger sign of _________ as requiring a visit to a health facility?   |  |  | **Never** | **Sometimes** | **Always** | | --- | --- | --- | --- | --- | | **a.** | Vaginal bleeding | 1 | 2 | 3 | | **b.** | Severe swelling | 1 | 2 | 3 | | **c.** | Fever | 1 | 2 | 3 | | **d.** | Other: **e.** _________________ | 1 | 2 | 3 |   **C39.** If the woman is bleeding after delivery, do you…   |  |  | **Yes** | **No** | | --- | --- | --- | --- | | **a.** | massage the uterus? | 1 | 2 | | **b.** | give misoprostol or oxytocin? | 1 | 2 | | **c.** | refer the patient to a clinic or a hospital? | 1 | 2 | |
| --- | --- | --- | --- | --- | --- | --- | --- | --- | --- | --- | --- | --- | --- | --- | --- | --- | --- | --- | --- | --- | --- | --- | --- | --- | --- | --- | --- | --- | --- | --- | --- | --- | --- | --- | --- | --- | --- | --- | --- | --- | --- | --- | --- | --- | --- | --- | --- | --- | --- | --- | --- | --- | --- | --- | --- | --- | --- | --- | --- | --- | --- | --- | --- | --- | --- | --- | --- | --- | --- | --- | --- | --- | --- | --- | --- | --- | --- | --- | --- | --- | --- | --- | --- | --- | --- | --- | --- | --- | --- | --- | --- | --- | --- | --- | --- | --- | --- | --- | --- | --- | --- | --- |
| **C40**. Do you know if the woman is bleeding too much…   |  |  | **Yes** | **No** | | --- | --- | --- | --- | | **a.** | by the volume of blood? | 1 | 2 | | **b.** | by watching how much bleeding? | 1 | 2 | | **c.** | by measuring the amount of blood loss? | 1 | 2 | | **d.** | by measuring her hemoglobin? | 1 | 2 | | **e.** | by looking for low blood pressure? | 1 | 2 |   **C41.** Is there a certain amount of time before you think labor is too long?  1 YES  2 NO 🡪 **GO TO QUESTION C43**  **C42.** Is it…  1 12 hours?  2 24 hours?  3 48 hours?  **C43.** In what position is the mother most commonly placed for the birth?  Is it…  1 Horizontal (litotomy)?  2 Vertical? | **C44.** Do you perform episiotomy?  1 YES  2 NO 🡪 **GO TO QUESTION C46**  **C45.** (IF YES) Is it…  1 midline?  2 side?  **C46.** If the mother has an episiotomy or vaginal tear, do you know how to repair it?    1 YES  2 NO 🡪 **GO TO QUESTION C48**  **C47.** (IF YES) Do you have a needle or needle holder and material?  1 YES  2 NO |

**C48.** Would you consider ___________ to be a sign that the mother or fetus is in trouble during birth?

|  | | **Yes** | **No** |  |  | **Yes** | **No** |
| --- | --- | --- | --- | --- | --- | --- | --- |
| **a.** | Too much bleeding | 1 | 2 |  | **a1**. (IF YES) Do you have a drape or other measure of blood loss? | 1 | 2 |
| **b.** | Labor is too long | 1 | 2 |  | **b1**. (IF YES) Do you use or have a clock? | 1 | 2 |
|  |  |  |  |  | **b2.** Do you use or have a partograph? | 1 | 2 |
| **c.** | Mother has seizures | 1 | 2 |  | **c1**. (IF YES) Do you know how to identify seizures? | 1 | 2 |
| **d.** | Mother has a fever | 1 | 2 |  | **d1**. (IF YES) Do you have a thermometer to take temperature? | 1 | 2 |
| **e.** | No fetal heart rate | 1 | 2 |  |  |  |  |
| **f.** | No fetal movement | 1 | 2 |  | **f1**. (IF YES) How do you detect fetal movement? _____________________________ | | |

| **C49.** Let’s talk about how you take care of the baby immediately after birth. Would you say you never, sometimes, or always _________?   |  |  | **Never** | **Sometimes** | **Always** | | --- | --- | --- | --- | --- | | **a.** | place baby on the mother’s abdomen? | 1 | 2 | 3 | | **b.** | place baby on a table or cloth? | 1 | 2 | 3 | | **c.** | clean mouth of baby with clean cloth or gauze? | 1 | 2 | 3 | | **d.** | suction mouth with a bulb? | 1 | 2 | 3 | | **e.** | dry baby’s head and body? | 1 | 2 | 3 | | **f.** | stimulate baby by spanking or shaking? | 1 | 2 | 3 | | **g.** | place on mother’s breast within first hour of life? | 1 | 2 | 3 | | **h.** | give teas or other liquids to baby? | 1 | 2 | 3 |   **C50.** Would you say that you never, sometimes, or always recognize that a baby is in trouble when the baby ________?   |  |  | **Never** | **Sometimes** | **Always** | | --- | --- | --- | --- | --- | | **a.** | Does not move | 1 | 2 | 3 | | **b.** | Is blue in the face or arms and legs | 1 | 2 | 3 | | **c.** | Has difficulty breathing | 1 | 2 | 3 | | **d.** | Has fever | 1 | 2 | 3 | | **e.** | Does not take milk from the breast | 1 | 2 | 3 | | **f.** | Shows slowing of heart rate | 1 | 2 | 3 | | **INTERVIEWER ASK THE FOLLOWING BEFORE GOING TO QUESTION C51:**  **C50g.** If the answer to C50d is sometimes or always, ask: How do you know if the baby has a fever? (length will be 255 characters)___________________  **C50h.** If the answer to C50e is sometimes or always, ask: How do you know that the baby has not taken milk? (length will be 255 characters)___________  **C50i.** If the answer to C50f is sometimes or always, ask: How do you know that the heart rate is slow? (length will be 255 characters)________________  **C51**. For women you deliver, would you say that you never, sometimes, or always have a follow-up visit to the mother and baby on the first or second day after birth?  1 NEVER  2 SOMETIMES  3 ALWAYS  **C52**. For women you deliver, how many postnatal visits do you routinely carry out?  1 NONE  2 1 to 3  3 4 to 9  4 10 OR MORE |
| --- | --- | --- | --- | --- | --- | --- | --- | --- | --- | --- | --- | --- | --- | --- | --- | --- | --- | --- | --- | --- | --- | --- | --- | --- | --- | --- | --- | --- | --- | --- | --- | --- | --- | --- | --- | --- | --- | --- | --- | --- | --- | --- | --- | --- | --- | --- | --- | --- | --- | --- | --- | --- | --- | --- | --- | --- | --- | --- | --- | --- | --- | --- | --- | --- | --- | --- | --- | --- | --- | --- | --- | --- | --- | --- | --- | --- | --- | --- | --- | --- | --- |

| **C53**. When discussing nutrition of the newborn with the mother, would you say that you never, sometimes, or always recommend  __________?   |  |  | **Never** | **Sometimes** | **Always** | | --- | --- | --- | --- | --- | | **a.** | Feeding colostrum | 1 | 2 | 3 | | **b.** | Starting immediate breastfeeding | 1 | 2 | 3 | | **c.** | Delaying breastfeeding until later on first day | 1 | 2 | 3 | | **d.** | Delaying breastfeeding until second day or later | 1 | 2 | 3 | | **e.** | exclusive breastfeeding | 1 | 2 | 3 | | **f.** | early introduction of other foods | 1 | 2 | 3 |   **C54.** Have you ever used a bag and mask to resuscitate a newborn?  1 YES  2 NO  **C55**. Have you been trained in its use?  1 YES  2 NO  **C56**. Would you say you never, sometimes, or always have a bag and mask available when you attend a birth?  1 NEVER  2 SOMETIMES  3 ALWAYS | **C57**. If you do not routinely have a bag and mask at deliveries, is it because… (MARK ALL THAT APPLY)  a you are not trained in its use?  b none is available?  c you don’t think it’s useful?  d it is not allowed by government?  e some other reason 🡪 Specify: **f.**  _____________________________________________  **C58**. Would you say you never, sometimes, or always have a clean birth kit with a razor/scissors, clamp, and cloth available for women you deliver?  1 NEVER  2 SOMETIMES  3 ALWAYS  **C59**. If you have a kit, is it supplied by…  1 government?  2 non-profit or private organization?  3 myself?  4 mother?  5 Some other way? 🡪 Specify: **6**._______________________  **C60**. Would you say that the mother or family have to pay for her own delivery kit?  1 NEVER  2 SOMETIMES  3 ALWAYS |
| --- | --- | --- | --- | --- | --- | --- | --- | --- | --- | --- | --- | --- | --- | --- | --- | --- | --- | --- | --- | --- | --- | --- | --- | --- | --- | --- | --- | --- | --- | --- | --- | --- | --- | --- | --- | --- |

**Section D. Reporting and Referrals**

| D01. Do you keep some information or log of pregnant women and babies you deliver?  1 YES  2 NO 🡪 **GO TO QUESTION D03**  D02. Do you keep information or log on… (MARK ALL THAT APPLY AND THEN GO TO QUESTION D04.)  a Mother’s age?  b Mother’s total number of pregnancies?  c Mother needed to go to hospital?  d Mother went to the hospital?  e Mother died?  f Delivery complications?  g Baby was born alive?  h Baby was born dead?  i Baby died after birth?  j Baby had birth defect?  k Baby needed to go to hospital?  L Baby was born before due date or premature?  m Other information: **n.** _______________________________ | D03. Is the reason that you are not keeping information or log because…  (MARK ALL THAT APPLY.)  a you cannot read or write?  b there are government sanctions against it?  c forms or logbook are not available?  d there was no way to keep the information, even though you wanted to?  e it is not required by the government, such as the Ministry of Health (MOH)?  f it will not be used or no one is interested?  g Other reason?: **h**.___________________________________  D04. Think about the information you report to a local, regional or national government, such as the MOH or a government health center. Would you say you never, sometimes, or always report ________?   |  |  | **Never** | **Sometimes** | **Always** | | --- | --- | --- | --- | --- | | **a.** | Live birth | 1 | 2 | 3 | | **b.** | Stillbirth | 1 | 2 | 3 | | **c.** | Miscarriage | 1 | 2 | 3 | | **d.** | Maternal death | 1 | 2 | 3 | | **e.** | Neonatal death | 1 | 2 | 3 |   D05. Does a government health official oversee your practice?  1 YES  2 NO  3 Don't Know |
| --- | --- | --- | --- | --- | --- | --- | --- | --- | --- | --- | --- | --- | --- | --- | --- | --- | --- | --- | --- | --- | --- | --- | --- | --- | --- | --- | --- | --- | --- | --- | --- |

| D06. If a woman has problems during pregnancy, labor or after delivery or if the baby is in trouble after delivery, can you make a referral to a health clinic or hospital? This can be by cell phone, land phone or some other way.  1 YES  2 NO 🡪 **GO TO QUESTION D08**  3 MAYBE  D07. Have you ever made a referral of mother or baby to a health clinic or hospital under emergency condition?  1 YES  2 NO  D08. Do you have an established method to get a sick mother or baby to a health clinic or hospital?  1 YES  2 NO  D09. In general, does a mother need to have cash-in-hand/money to receive care at a health clinic or hospital?  1 YES  2 NO  D10. Is permission from the father or in-laws required for transporting the mother or the baby to a health clinic or hospital?  1 YES  2 NO | D11. Think of the health clinic or hospital you usually make referrals to. Have you ever been inside the facility?  1 YES  2 NO 🡪 **GO TO QUESTION D14**  D12. In general, do you feel that this health clinic or hospital provided adequate health care?  1 YES 🡪 **GO TO QUESTION D14**  2 NO  D13. Is it because… (MARK ALL THAT APPLY)  a Women or baby were not examined quickly?  b Women or baby were not treated well?  c Other d. _______________________________________  D14. Would you say you never, sometimes, or always receive follow-up contact regarding your referrals from the doctor or nurse at this health clinic or hospital?  1 NEVER  2 SOMETIMES  3 ALWAYS  D15. Would you make a referral again to this health clinic or hospital?  1 YES  2 NO |
| --- | --- |

**KENYA SITE SPECIFIC QUESTIONS**

1. Have you participated in Helping Babies Breathe:

Initial Training?  Yes  No

Refresher Training?  Yes  No

2. Do you have gloves?  Yes  No

3. What do you apply on the cord after cutting?  Alcohol  Gentian Violet (GV)  Other _______

4. Have you used mouth-to-mouth to resuscitate a newborn?  Yes  No

5. Have you used traditional medications/herbs during pregnancy or labor?  Yes  No

If yes, for what indication have you used these medications?

Prolonged/Obstructed labor

Retained placenta

Bleeding

Fever

Other _________

6. Do you charge for your services?  Always  Sometimes  Never

7. If yes, do you charge in:

Local Currency: Indicate amount ________

Barter Item

Other ________

8. How do you manage retained placenta after delivery?

Manual extraction

Uterine massage

Oxytocin

Refer to health facility

Other _______

9. Do you report your pregnancy and delivery outcomes to the chief/village elder?  Yes  No

10. Have you made referrals to health facilities for the following indications? (Check all that apply):

Obstructed labor

Prolonged labor

Bleeding

Retained placenta

Cord prolapsed

Prematurity

Failure to detect fetal heart rate

Mother with hypertension

Mother with seizures

Breech presentation

Baby not breathing after delivery

Other ______

11. How would you rate the following experiences with the referral health facility staff?

Very Good Good Fair Poor Very Poor

Friendliness

Communication/Feedback

Interaction with families
