# Supplementary material for: Self-reported practices among traditional birth attendants surveyed in western Kenya: a descriptive study
Source: BMC Pregnancy Childbirth. 2016 Aug 12;16:219. doi: 10.1186/s12884-016-1007-8 (PMC4981994; doi:10.1186/s12884-016-1007-8)
Supplement: Additional file 2: — Frequency tables for self-reported practices of TBAs in Western Kenya from which the results and findings for the descriptive study reported in this manuscript are derived. (PDF 40 kb) [file 12884_2016_1007_MOESM2_ESM.pdf]

```
FREQUENCIES VARIABLES=GNCluster HBBTrainInt HBBTrainRef Gloves CordAlcohol Cor
dGV CordOther CordOtherDes Mouth2Mouth TradRXEver TradRXObsLabor TradRxPlacent
a TradRxBleed TradRxFever TradRxOther TradRxOtherText PayforTBA TBAPayKSH KSHC
ost TBAPayBarter
TBAPayOther PlacManual PlacMassage PlacOxytocin PlacRefer PlacOther PlacOtherT
ext Report2Elder ReferObsLabor ReferProLabor ReferBleed ReferRetainPlac ReferC
ordProlapse ReferPreterm ReferNoFetalHR ReferMomBP ReferMomSeize ReferBreech R
eferBabyAsphy
ReferOther ReferOtherDesc HCFriendly HCCommunicate HCFamInteract
  /ORDER=ANALYSIS.
```

Frequencies

```
[DataSet1] C:\Documents and Settings\shbucher\Desktop\Old PC\shbucher\Global N
etwork Kenya-specific research\TBA survey\SPSS data analysis\CLEAN_Kenya only
CBA survey data.sav
```

Statistics

		GNCluster	Receive HBB initial training?	Receive HBB refresher training?	Do you have gloves?	Apply alchohol to cord
N	Valid	101	101	101	101	101
	Missing	0	0	0	0	0

Statistics

		Apply gentian violet to cord	Other reported cord care	Description	Have you used mouth to mouth to resuscitate a baby?	Ever used TradRx/herbs for pregnant/laboring moms?
N	Valid	101	101	101	101	101
	Missing	0	0	0	0	0

Statistics

		Used TradRx/herbs for obs/prolonged labor.	Used TradRx/herbs for retained placenta	Used TradRx/herbs for bleeding	Used TradRx/herbs for fever	Other reported use of TradRx/herbs
N	Valid	101	101	101	101	101
	Missing	0	0	0	0	0

Statistics

		Description of other herb use	Do you charge for TBA services?	TBA charges in shillings	Average TBA charge in KSH	Accept barter for TBA services?
N	Valid	101	101	101	100	101
	Missing	0	0	0	1	0

Statistics

		Other reported ways to pay for TBA services	Manage retained placenta: manual extraction	Manage retained placenta: uterine massage	Manage retained placenta: Oxytocin	Manage retained placenta: refer to health facility
N	Valid	101	101	101	101	101
	Missing	0	0	0	0	0

Statistics

		Manage retained placenta: other reported	Other methods manage retained placenta	Report outcomes to elder/chief?	Ever referred to health facility for obstructed labor	Ever referred to health facility for prolonged labor
N	Valid	101	101	101	101	101
	Missing	0	0	0	0	0

Statistics

		Ever referred to health facility for bleeding	Ever referred to health facility for retained placenta	Ever referred to health facility for prolapsed cord	Ever referred to health facility for prematurity	Ever referred to health facility for no fetal heart rate
N	Valid	101	101	101	101	101
	Missing	0	0	0	0	0

Statistics

		Ever referred to health facility for mom hypertension	Ever referred to health facility for mom seizures	Ever referred to health facility for breech	Ever refer to health facility for baby not breathe	Ever refer for other conditions
N	Valid	101	101	101	101	101
	Missing	0	0	0	0	0

Statistics

		Other reported referral conditions	HCFriendly	HCCommunicate	HCFamInteract
N	Valid	101	100	101	101
	Missing	0	1	0	0

Frequency Table

GNCluster

		Frequency	Percent	Valid Percent	Cumulative Percent
Valid	Alupe	6	5.9	5.9	5.9
	Makunga	7	6.9	6.9	12.9
	Matayos	6	5.9	5.9	18.8
	Moding	6	5.9	5.9	24.8
	Mumias	6	5.9	5.9	30.7
	Nambale	6	5.9	5.9	36.6
	Nasewa	5	5.0	5.0	41.6
	Sirisia	6	5.9	5.9	47.5
	Amukura	7	6.9	6.9	54.5
	Bokoli	4	4.0	4.0	58.4
	Bumula	7	6.9	6.9	65.3
	Bumala B	6	5.9	5.9	71.3
	Chwele	7	6.9	6.9	78.2
	Khunyangu	7	6.9	6.9	85.1
	Kocholia	7	6.9	6.9	92.1
	Lukolis	6	5.9	5.9	98.0
	Naswea	1	1.0	1.0	99.0
	Unknown	1	1.0	1.0	100.0
	Total	101	100.0	100.0	

Receive HBB initial training?

		Frequency	Percent	Valid Percent	Cumulative Percent
Valid	No	77	76.2	76.2	76.2
	Yes	23	22.8	22.8	99.0
	Missing	1	1.0	1.0	100.0
	Total	101	100.0	100.0	

Receive HBB refresher training?

		Frequency	Percent	Valid Percent	Cumulative Percent
Valid	No	98	97.0	97.0	97.0
	Yes	2	2.0	2.0	99.0
	Missing	1	1.0	1.0	100.0
	Total	101	100.0	100.0	

Do you have gloves?

		Frequency	Percent	Valid Percent	Cumulative Percent
Valid	No	3	3.0	3.0	3.0
	Yes	98	97.0	97.0	100.0
	Total	101	100.0	100.0	

Apply alchohol to cord

		Frequency	Percent	Valid Percent	Cumulative Percent
Valid	No	99	98.0	98.0	98.0
	Missing	2	2.0	2.0	100.0
	Total	101	100.0	100.0	

Apply gentian violet to cord

		Frequency	Percent	Valid Percent	Cumulative Percent
Valid	No	99	98.0	98.0	98.0
	Missing	2	2.0	2.0	100.0
	Total	101	100.0	100.0	

Other reported cord care

		Frequency	Percent	Valid Percent	Cumulative Percent
Valid	No	46	45.5	45.5	45.5
	Yes	53	52.5	52.5	98.0
	Missing	2	2.0	2.0	100.0
	Total	101	100.0	100.0	

Description

		Frequency	Percent	Valid Percent	Cumulative Percent
Valid	Jik (bleach)	1	1.0	1.0	1.0
	Missing	2	2.0	2.0	3.0
	Nothing	44	43.6	43.6	46.5
	Oil	2	2.0	2.0	48.5
	Powder	15	14.9	14.9	63.4
	Powder, Oil	2	2.0	2.0	65.3
	Spirit	35	34.7	34.7	100.0
	Total	101	100.0	100.0	

Have you used mouth to mouth to resuscitate a baby?

		Frequency	Percent	Valid Percent	Cumulative Percent
Valid	No	65	64.4	64.4	64.4
	Yes	23	22.8	22.8	87.1
	Missing	13	12.9	12.9	100.0
	Total	101	100.0	100.0	

Ever used TradRx/herbs for pregnant/laboring moms?

		Frequency	Percent	Valid Percent	Cumulative Percent
Valid	No	82	81.2	81.2	81.2
	Yes	19	18.8	18.8	100.0
	Total	101	100.0	100.0	

Used TradRx/herbs for obs/prolonged labor.

		Frequency	Percent	Valid Percent	Cumulative Percent
Valid	No	89	88.1	88.1	88.1
	Yes	2	2.0	2.0	90.1
	Missing	10	9.9	9.9	100.0
	Total	101	100.0	100.0	

Used TradRx/herbs for retained placenta

		Frequency	Percent	Valid Percent	Cumulative Percent
Valid	No	86	85.1	85.1	85.1
	Yes	9	8.9	8.9	94.1
	Missing	6	5.9	5.9	100.0
	Total	101	100.0	100.0	

Used TradRx/herbs for bleeding

		Frequency	Percent	Valid Percent	Cumulative Percent
Valid	No	88	87.1	87.1	87.1
	Yes	3	3.0	3.0	90.1
	Missing	10	9.9	9.9	100.0
	Total	101	100.0	100.0	

Used TradRx/herbs for fever

		Frequency	Percent	Valid Percent	Cumulative Percent
Valid	No	90	89.1	89.1	89.1
	Yes	1	1.0	1.0	90.1
	Missing	10	9.9	9.9	100.0
	Total	101	100.0	100.0	

Other reported use of TradRx/herbs

		Frequency	Percent	Valid Percent	Cumulative Percent
Valid	No	90	89.1	89.1	89.1
	Yes	1	1.0	1.0	90.1
	Missing	10	9.9	9.9	100.0
	Total	101	100.0	100.0	

Description of other herb use

		Frequency	Percent	Valid Percent	Cumulative Percent
Valid		100	99.0	99.0	99.0
	Lack of appetite	1	1.0	1.0	100.0
	Total	101	100.0	100.0	

Do you charge for TBA services?

		Frequency	Percent	Valid Percent	Cumulative Percent
Valid	Never	2	2.0	2.0	2.0
	Sometimes	48	47.5	47.5	49.5
	Always	48	47.5	47.5	97.0
	Missing	3	3.0	3.0	100.0
	Total	101	100.0	100.0	

TBA charges in shillings

		Frequency	Percent	Valid Percent	Cumulative Percent
Valid	No	3	3.0	3.0	3.0
	Yes	97	96.0	96.0	99.0
	Missing	1	1.0	1.0	100.0
	Total	101	100.0	100.0	

Average TBA charge in KSH

		Frequency	Percent	Valid Percent	Cumulative Percent
Valid	0	3	3.0	3.0	3.0
	20	1	1.0	1.0	4.0
	30	1	1.0	1.0	5.0
	50	2	2.0	2.0	7.0
	90	1	1.0	1.0	8.0
	100	20	19.8	20.0	28.0
	150	1	1.0	1.0	29.0
	200	37	36.6	37.0	66.0
	250	1	1.0	1.0	67.0
	300	18	17.8	18.0	85.0
	400	2	2.0	2.0	87.0
	500	11	10.9	11.0	98.0
	600	1	1.0	1.0	99.0
	700	1	1.0	1.0	100.0
	Total	100	99.0	100.0	
Missing	System	1	1.0		
Total		101	100.0		

Accept barter for TBA services?

		Frequency	Percent	Valid Percent	Cumulative Percent
Valid	No	80	79.2	79.2	79.2
	Yes	20	19.8	19.8	99.0
	Missing	1	1.0	1.0	100.0
	Total	101	100.0	100.0	

Other reported ways to pay for TBA services

		Frequency	Percent	Valid Percent	Cumulative Percent
Valid		82	81.2	81.2	81.2
	Chicken	14	13.9	13.9	95.0
	Chicken, maize	1	1.0	1.0	96.0
	Lunch	1	1.0	1.0	97.0
	Maize	1	1.0	1.0	98.0
	Maize, Cassava	1	1.0	1.0	99.0
	Sugar, tea leave	1	1.0	1.0	100.0
	Total	101	100.0	100.0	

Manage retained placenta: manual extraction

		Frequency	Percent	Valid Percent	Cumulative Percent
Valid	No	85	84.2	84.2	84.2
	Yes	15	14.9	14.9	99.0
	Missing	1	1.0	1.0	100.0
	Total	101	100.0	100.0	

Manage retained placenta: uterine massage

		Frequency	Percent	Valid Percent	Cumulative Percent
Valid	No	53	52.5	52.5	52.5
	Yes	47	46.5	46.5	99.0
	Missing	1	1.0	1.0	100.0
	Total	101	100.0	100.0	

Manage retained placenta: Oxytocin

		Frequency	Percent	Valid Percent	Cumulative Percent
Valid	No	94	93.1	93.1	93.1
	Yes	6	5.9	5.9	99.0
	Missing	1	1.0	1.0	100.0
	Total	101	100.0	100.0	

Manage retained placenta: refer to health facility

		Frequency	Percent	Valid Percent	Cumulative Percent
Valid	No	39	38.6	38.6	38.6
	Yes	61	60.4	60.4	99.0
	Missing	1	1.0	1.0	100.0
	Total	101	100.0	100.0	

Manage retained placenta: other reported

		Frequency	Percent	Valid Percent	Cumulative Percent
Valid	No	84	83.2	83.2	83.2
	Yes	16	15.8	15.8	99.0
	Missing	1	1.0	1.0	100.0
	Total	101	100.0	100.0	

Other methods manage retained placenta

		Frequency	Percent	Valid Percent	Cumulative Percent
Valid		85	84.2	84.2	84.2
	Cooking stick down throat	2	2.0	2.0	86.1
	Cooking stick or chain of beads down mom's throat	1	1.0	1.0	87.1
	Give mother hot porridge	1	1.0	1.0	88.1
	Herbal oxytocin	2	2.0	2.0	90.1
	Herbs	6	5.9	5.9	96.0
	Lantana leaves	1	1.0	1.0	97.0
	Nipple massage	1	1.0	1.0	98.0
	Put beads in mom's throat; rubs a stone on spine	1	1.0	1.0	99.0
	Rub grinding stone on mother's back	1	1.0	1.0	100.0
	Total	101	100.0	100.0	

Report outcomes to elder/chief?

		Frequency	Percent	Valid Percent	Cumulative Percent
Valid	No	13	12.9	12.9	12.9
	Yes	84	83.2	83.2	96.0
	Missing	4	4.0	4.0	100.0
	Total	101	100.0	100.0	

Ever referred to health facility for obstructed labor

		Frequency	Percent	Valid Percent	Cumulative Percent
Valid	No	10	9.9	9.9	9.9
	Yes	91	90.1	90.1	100.0
	Total	101	100.0	100.0	

Ever referred to health facility for prolonged labor

		Frequency	Percent	Valid Percent	Cumulative Percent
Valid	No	13	12.9	12.9	12.9
	Yes	88	87.1	87.1	100.0
	Total	101	100.0	100.0	

Ever referred to health facility for bleeding

		Frequency	Percent	Valid Percent	Cumulative Percent
Valid	No	7	6.9	6.9	6.9
	Yes	94	93.1	93.1	100.0
	Total	101	100.0	100.0	

Ever referred to health facility for retained placenta

		Frequency	Percent	Valid Percent	Cumulative Percent
Valid	No	16	15.8	15.8	15.8
	Yes	85	84.2	84.2	100.0
	Total	101	100.0	100.0	

Ever referred to health facility for prolapsed cord

		Frequency	Percent	Valid Percent	Cumulative Percent
Valid	No	9	8.9	8.9	8.9
	Yes	92	91.1	91.1	100.0
	Total	101	100.0	100.0	

Ever referred to health facility for prematurity

		Frequency	Percent	Valid Percent	Cumulative Percent
Valid	No	20	19.8	19.8	19.8
	Yes	81	80.2	80.2	100.0
	Total	101	100.0	100.0	

Ever referred to health facility for no fetal heart rate

		Frequency	Percent	Valid Percent	Cumulative Percent
Valid	No	45	44.6	44.6	44.6
	Yes	56	55.4	55.4	100.0
	Total	101	100.0	100.0	

Ever referred to health facility for mom hypertension

		Frequency	Percent	Valid Percent	Cumulative Percent
Valid	No	77	76.2	76.2	76.2
	Yes	24	23.8	23.8	100.0
	Total	101	100.0	100.0	

Ever referred to health facility for mom seizures

		Frequency	Percent	Valid Percent	Cumulative Percent
Valid	No	19	18.8	18.8	18.8
	Yes	82	81.2	81.2	100.0
	Total	101	100.0	100.0	

Ever referred to health facility for breech

		Frequency	Percent	Valid Percent	Cumulative Percent
Valid	No	30	29.7	29.7	29.7
	Yes	71	70.3	70.3	100.0
	Total	101	100.0	100.0	

Ever refer to health facility for baby not breathe

		Frequency	Percent	Valid Percent	Cumulative Percent
Valid	No	19	18.8	18.8	18.8
	Yes	82	81.2	81.2	100.0
	Total	101	100.0	100.0	

Ever refer for other conditions

		Frequency	Percent	Valid Percent	Cumulative Percent
Valid	No	87	86.1	86.1	86.1
	Yes	14	13.9	13.9	100.0
	Total	101	100.0	100.0	

Other reported referral conditions

		Frequency	Percent	Valid Percent	Cumulative Percent
Valid		96	95.0	95.0	95.0
	Big baby	1	1.0	1.0	96.0
	Goiter	1	1.0	1.0	97.0
	Unable to push	1	1.0	1.0	98.0
	Yellow baby	2	2.0	2.0	100.0
	Total	101	100.0	100.0	

HCFriendly

		Frequency	Percent	Valid Percent	Cumulative Percent
Valid	Very Poor	7	6.9	7.0	7.0
	Poor	5	5.0	5.0	12.0
	Fair	17	16.8	17.0	29.0
	Good	69	68.3	69.0	98.0
	Very Good	2	2.0	2.0	100.0
	Total	100	99.0	100.0	
Missing	System	1	1.0		
Total		101	100.0		

HCCommunicate

		Frequency	Percent	Valid Percent	Cumulative Percent
Valid	Very poor	7	6.9	6.9	6.9
	Poor	5	5.0	5.0	11.9
	Fair	18	17.8	17.8	29.7
	Good	68	67.3	67.3	97.0
	Very Good	3	3.0	3.0	100.0
	Total	101	100.0	100.0	

HCFamInteract

		Frequency	Percent	Valid Percent	Cumulative Percent
Valid	Very poor	7	6.9	6.9	6.9
	Poor	5	5.0	5.0	11.9
	Fair	19	18.8	18.8	30.7
	Good	68	67.3	67.3	98.0
	Very Good	2	2.0	2.0	100.0
	Total	101	100.0	100.0	

```
FREQUENCIES VARIABLES=GNCluster TradRXEver TradRXObsLabor TradRxPlacenta TradR
xBleed TradRxFever TradRxOther TradRxOtherText PayforTBA TBAPayKSH KSHCost TBA
PayBarter TBAPayOther HCFamInteract HCFriendly HCCommunicate
  /ORDER=ANALYSIS.
GET
  FILE='C:\Documents and Settings\shbucher\Desktop\Old PC\shbucher\Global Netw
ork Kenya-specific research\TBA survey\SPSS data analysis\CLEAN_Kenya only CBA
 survey data.sav'.
DATASET NAME DataSet1 WINDOW=FRONT.
```
